# The critical roles of histone deacetylase 3 in the pathogenesis of solid organ injury

**DOI:** 10.1038/s41419-021-04019-6

**Published:** 2021-07-23

**Authors:** Li Ning, Xiong Rui, Wang Bo, Geng Qing

**Affiliations:** Department of Thoracic Surgery, Renmin Hospital of Wuhan University, 430060 Wuhan, China

**Keywords:** Enzyme mechanisms, Pharmacology

## Abstract

Histone deacetylase 3 (HDAC3) plays a crucial role in chromatin remodeling, which, in turn, regulates gene transcription. Hence, HDAC3 has been implicated in various diseases, including ischemic injury, fibrosis, neurodegeneration, infections, and inflammatory conditions. In addition, HDAC3 plays vital roles under physiological conditions by regulating circadian rhythms, metabolism, and development. In this review, we summarize the current knowledge of the physiological functions of HDAC3 and its role in organ injury. We also discuss the therapeutic value of HDAC3 in various diseases.

## Introduction

Solid-organ injury is among the leading causes of death globally and significantly impacts the quality of life. Solid-organ injury can be acute injury occurring in the perioperative period or chronic injury caused by long-term stimulation and toxic insult. Acute injury includes myocardial, cerebral, renal, and hepatic ischemia-reperfusion injury (IRI) [1, 2]. Acute organ injury is characterized by potent proinflammatory responses involving leukocyte migration, cytokine release, microvascular thromboses, and cell death [3–5]. The proinflammatory phase of acute injury-associated systemic immune responses is often followed by an immunosuppressive phase [6]. During the pro-inflammatory phase, chemokines and cytokines are secreted by immune cells, and damage-associated molecular patterns (DAMPs) activate pattern recognition receptors (PRRs) [7]. Chronic injury is typically associated with metabolic rewiring, immune imbalance, and tissue remodeling [8, 9]. Most patients with organ injury receive temporary organ support or replacement therapies as there are no specific treatments to reverse or maintain individual organ functions [10]. Therefore, preventive and early supportive interventions are needed.

**Fig. 1:**
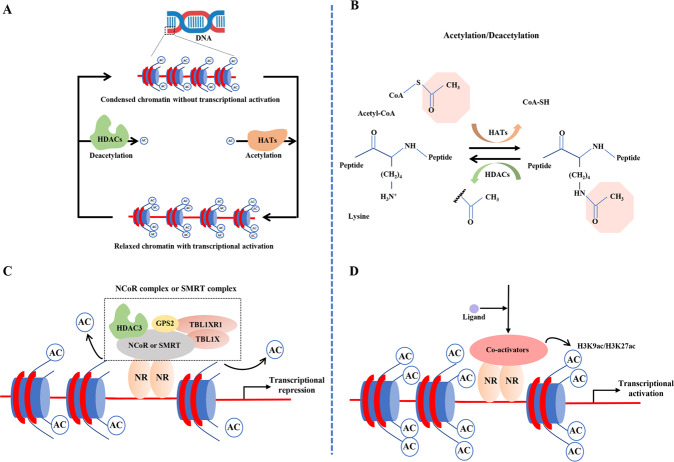
Schematic representation of the histone acetylation and deacetylation by HATs and HDACs. **A** Condensation and relaxation of chromatin due to histone deacetylation and acetylation, respectively. Histone acetylation levels are determined by the interplay between HATs and HDACs. Activation of HDACs leads to a net decrease of histone acetylation, chromatin condensation, and transcriptional repression. Activation of HATs results in a net increase of histone acetylation, chromatin relaxation, and transcriptional activation. **B** The chemical formula of histone acetylation and deacetylation. **C** Nuclear receptor co-repressor complexes containing HDAC3, GPS2, TBLX, and TBL1XR bind to nuclear receptors without ligands to induce transcriptional repression via histone deacetylation. **D** Nuclear receptor-mediated ligand binding inhibits the co-repressor complex and recruits co-activators, facilitating histone acetylation and gene transcription.

Epigenetic modifications regulate gene expression and developmental programs in the absence of changes in the gene sequence. Epigenetic modifications include histone modification, DNA methylation, chromosome remodeling, and regulation of transcription or translation by non-coding RNAs [11]. Histone acetylation and deacetylation have been extensively studied in recent years. Histones are intra-nuclear cationic proteins expressed in eukaryotic cells. They modulate gene expression by stabilizing chromatin structure; hence, alterations in histone patterns have been implicated in various diseases [12]. Histone acetylation was first described by Allfrey V in 1964 [13]. By regulating the acetylation of the N-terminal lysine residues of histones, histone acetyltransferases (HATs) and histone deacetylases (HDACs) determine chromatin structure and gene expression (Fig. 1A) [14]. Mounting evidence suggests that HDAC3 plays a key role in solid organ injury [15–17]. In this review article, we provide an overview of the current knowledge of the role of HDAC3 in the pathogenesis of solid organ injury, focusing on the possible underlying molecular mechanisms.

## HATs and HDACs

Histones are critical components of nucleosomes. Post-translational modifications of histones affect chromatin structure and gene expression [18]. Histones are tightly coiled by DNA to form nucleosomes, contributing to the highly compact packaging of the eukaryotic genome [19]. The positively charged octamers of a linker histone (H1 or H5) and four highly basic histones (H3, H4, H2A, and H2B) interact with the negatively charged DNA via electrostatic bonds. H1 and H5 are responsible for the stabilization of chromosomes, promoting the formation of higher-order structures. Each histone octamer is tightly coiled by 147 base pairs of DNA [20]. These highly compact structures are dynamic and can be regulated by the binding of transcription factors to promoter sequences within the genome [21]. Epigenetic modifications can serve as markers to identify the different types of chromatin. For instance, heterochromatin is characterized by low acetylation levels, whereas euchromatin contains relatively high acetylation levels. Abnormal acetylation of histones may dysregulation gene expression, contributing to the occurrence of diseases [22].

**Fig. 2:**
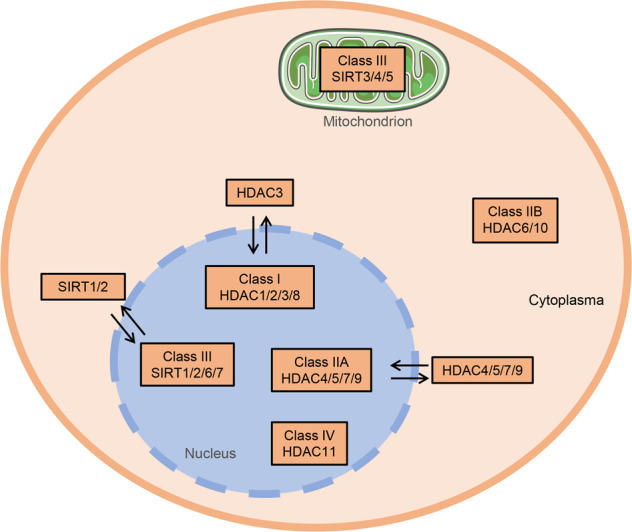
The cellular localization of the four classes of histone deacetylases. Class I, IIA, VI, and part of class III HDACs are mainly found in the nucleus. HDAC3, HDAC4, HDAC7, HDAC9, SIRT1, and SIRT2 shuffle between the nucleus and cytoplasm. Class IIB HDACs, including HDAC6 and HDAC10, are localized in the cytoplasm. Class III HDACs, including SIRT3, SIRT4, and SIRT5, are localized in mitochondria.

Histone acetylation and deacetylation levels are tightly regulated by the balance between the opposing activities of HATs and HDACs [23]. HATs are divided into three families: Gcn5-related acetyltransferases (GNATs); p300/CREB-binding proteins (CBP); and MOZ, Ybf2/Sas3, Sas2, and Tip60 (MYST)-related HATs. HATs catalyze lysine acetylation of histones by transferring the acetyl-CoA acetyl group to the ε-amino group of the internal lysine residue in the N-terminus. The addition of an acetyl group disrupts the electrostatic interaction between the DNA and histones. By neutralizing the positive lysine charge, histone acetylation alters chromatin structure and gene expression (Fig. 1B) [24, 25]. Deacetylation of histones is usually mediated by HDACs and promotes chromatin condensation and transcriptional repression [23]. In mammals, HDACs are divided into four categories: class I, II, III, and IV. Class I HDACs include HDAC1, HDAC2, HDAC3, and HDAC8. These HDACs are predominately found in the nucleus, but HDAC3 can translocate from the nucleus into the cytosol [26]. Class II HDACs include IIa HDACs (HDAC4, HDAC5, HDAC7, and HDAC9) and IIb HDACs (HDAC6 and HDAC10). Class IIa HDACs can shuttle between the nucleus and the cytosol. Class III HDACs are also known as sirtuins because of their homology to the yeast HDAC Sir2; this class includes SIRT1, SIRT2, SIRT3, SIRT4, SIRT5, SIRT6, and SIRT7. Class IV HDACs only includes one member (HDAC11), which is predominantly expressed in the nucleus (Fig. 2). Class III HDACs are NAD-dependent enzymes, whereas the other HDACs are zinc-dependent enzymes [27, 28].

## Structure and function of HDAC3

### The unique structure of HDAC3

All class I HDAC members except HDAC8 share a similar structure, especially near the substrate-binding site [29]. Structural differences also exist between HDAC3 and other class I HDACs. HDAC3 possesses an aspartate residue at position 92, whereas HDAC1 and HDAC2 have a glutamate residue at this position at the outer rim of the cavity. Furthermore, HDAC1 and HDAC2 have a tyrosine residue at position 199, whereas HDAC3 has phenylalanine. Particularly, position 107 of HDAC3 is a tyrosine, while in the same position of HDAC1/2 is serine. Tyrosine 107 of HDAC3 leads to steric hindrance for binding to the foot pocket, precluding the binding of larger functional groups to inhibitors. Based on these differences, selective inhibitors against HDAC1, HDAC2, and HDAC3 can be designed [30, 31]. Hydrophobic amino acids at positions 13 and 29 of HDAC3 also differ from those in HADC1 and HDAC2, enabling the development of selective inhibitors targeting the foot pocket [32]. The biological function of HDAC3 requires nuclear receptor co-repressors, including silencing mediator of retinoic acid and thyroid hormone receptor (SMRT or NCoR2) and nuclear receptor co-repressor 1 (NCoR1) [33, 34]. Theoretically, the members involving 1, 2, 3, and 8 in the class I HDAC family may share similar functions and other members of the class I HDAC family will compensate when HDAC3 is knocked out effect. However, previous studies demonstrated that the expression of HDAC3 is not in accordance with HDAC1, HDAC2, and HDAC8 in the context of different pathological stimuli. Meanwhile, other members also display different biological functions from HDAC3 [35–37]. Therefore, we speculate that the other members in class I HDAC will not compensate when HDAC3 is absent based on the published articles. Of course, the expression of HDAC1, 2 and 8 should be also detected when HDAC3 was silenced in future studies.

### Enzymatic activity and non-enzymatic functions of HDAC3

Nuclear receptors act as genetic switches regulating gene transcription by activating signal-dependent transcription factors. In turn, transcription factors integrate hormonal, metabolic, and environmental cues, recruiting various co-repressors and co-activators to specific genomic sequences [38]. HDAC3-containing nuclear receptor co-repressor complexes, including NCoR and SMRT, bind to ligand-free nuclear receptors, which directly repress gene expression. NCoR and SMRT complexes contain WD40 repeat-containing proteins, such as TBL1XR1 and TBL1X (Fig. 1C). These complexes recruit the 19S proteasome and the ubiquitylation machinery to histones [39]. G-protein pathway suppressor 2 (GPS2) is another core element of NCoR and SMRT complexes [40]; however, the role of GPS2 remains unclear. Noteworthily, under certain circumstances, HDAC3 indirectly activates gene expression. By contrast, nuclear receptor-mediated ligand binding inactivates the co-repressor complex and recruits co-activators, thereby facilitating gene transcription via histone acetylation [41] (Fig. 1D).

#### Enzymatic activity

The catalytic function of HDAC3 requires the physical interaction between HDAC3 and the deacetylase-activating domain (DAD) of NCoR and SMRT proteins. Crystal structure analysis of these complexes unveiled that abundant protein-protein interactions between the N-terminus of HDAC3 and the DAD of SMRT. Inositol tetraphosphate (Ins[1,4,5,6]P_4_ or IP4) has been identified, acting as “intermolecular glue” enhancing the interaction between HDAC3 and SMRT DAD via salt bridges and hydrogen bonds [42]. Intriguingly, once HDAC3 is dissociated from the NCoR or SMRT complex, it becomes unstable and is sequestered into a TCP1-ring complex in the cytoplasm [43]. TCP1 promotes HDAC3 folding in the cytoplasm, facilitating the formation of NCoR or SMRT complex containing an active HDAC3 enzyme in an ATP-dependent manner [44].

#### Non-enzymatic activity

In addition to its enzymatic functions, HDAC3 also displays non-enzymatic activities. Point mutations in Y298F, one of the active sites of HDAC3, disrupt its deacetylase activity. In the livers of HDAC3-deficient mice, the mutant HDAC3 can partially rescue hepatosteatosis and inhibit the expression of lipogenic genes, suggesting that HDAC3 has non-enzymatic functions [44]. Moreover, global HDAC3 knockout may lead to embryonic lethality because of gastrulation defects [45]. However, mutations in the DAD of SMRT and NCoR did not affect the Mendelian ratios of offspring mice, although the mice showed little HDAC3 enzymatic activity [45, 46]. Indeed, approximately 10% of enzymes with inactivating mutations in their active site are conserved in mammals, further supporting their non-catalytic functions [47]. Therefore, the non-enzymatic functions of HDAC3 should be taken into account in the development of HDAC3 inhibitors.

### Physiological functions of HDAC3

Several genetically engineered mice with cell type-specific deletion of the *Hdac3* gene have been developed recently to investigate the role of HDAC3. Notably, HDAC3 has been shown to regulate metabolism by increasing fatty acid oxidation and enhancing circadian histone deacetylation [47, 48]. HDAC3 also inhibits white adipose tissue metabolism by enhancing the futile cycle of fatty acid synthesis and oxidation and by decreasing acetylation in the enhancers of *Ucp1* and *Ppar-γ* [48]. In addition, HDAC3 is essential for the development of vital organs. For example, mice with lung endodermal epithelium-specific *Hdac3* knockout displayed lethality 2 to 10 days after birth due to defects in lung sacculation and early alveologenesis [49]. Similarly, cardiac progenitor cell-specific *Hdac3* knockout led to ventricular septal defects and underdevelopment of ventricular walls, causing embryonic lethality [46]. Apart from lung and heart development, HDAC3 is also essential for the development and remodeling of bones [50].

**Fig. 3:**
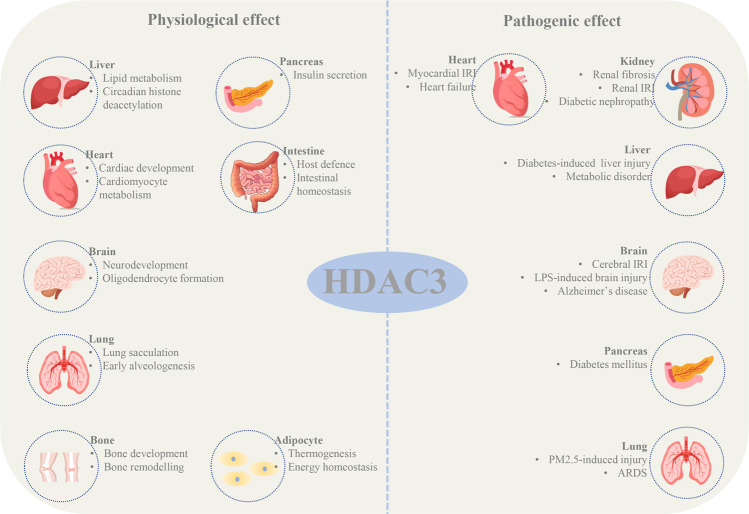
The physiological functions and pathogenic effects of HDAC3. Under physiological condition, HDAC3 is mainly responsible for the development and homeostasis of liver, heart, brain, lung, bone, pancreas, intestine, and adipocyte. However, the abnormal expression of HDAC3 also contributes to organ injury including heart, brain, pancreas, kidney, lung, and liver.

Moreover, HDAC3 contributes to the maintenance of intestinal homeostasis and host defense. Notably, HDAC3 loss can cause inflammation and intestinal damage [50]. Furthermore, HDAC3-deficiency in neural progenitor cells impaired cortical lamination and neuronal migration, resulting in death within 16 h after birth. The critical role of HDAC3 in neuronal cell fate and function may be associated with HDAC3-mediated expression of T-box brain protein 1 [51]. Based on these findings, we conclude that HDAC3 expression and function are essential for multiple aspects of mammalian physiology and homeostasis (Fig. 3). The role of HDAC3 in organ injury is described below (Table 1).

**Table 1:** Subcellular localization of HDAC3 and its roles in different disease models.

Models	Subcellular localization	Key mechanisms	Activity	Reference
Cerebral IRI	Shuttling between the nucleus and the cytosol in microglia	Deacetylating p65 at K122 in the cytosol and interacting with p65 in the nucleus to induce neuroinflammation by activating cGAS-STING axis	Enzymatic activity	[37]
Ischemic brain damage	Mainly concentrating in the nucleus in microglia 24 h following ischemia and gradually spreading to cytoplasm 72 h following ischemia	Deacetylating STAT1 and subsequently promoted STAT1 phosphorylation, contributing to brain damage via the regulation of AIM2	Enzymatic activity	[58]
Cerebral IRI in the diabetic state	Not detected	Promoting the cerebral infarct volume and cytotoxicity by upregulating Bmal1	Enzymatic activity	[61]
Ischemic stroke	Distributed both in the nucleus and cytoplasm in cortical neurons before ischemic preconditioning; distributed mainly in the cytoplasm after ischemic preconditioning treatment	Potentiating transcriptional initiation of oxidation relative genes involving *Hspa1a*, *Bcl2l1*, and *Prdx2*	Enzymatic activity	[133]
Chronic constriction injury	Mainly concentrating in the nucleus in the hippocampus	Deacetylating H3 and H4 in the hippocampus and triggering memory impairment	Enzymatic activity	[35]
Alzheimer’s disease	Both nucleus and the cytosol in HEK/APPsw cells.	Increasing Aβ_1–42_ accumulation and both tau acetylation and phosphorylation at disease residues, thus impairing learning and memory	Enzymatic activity	[36]
Acute lung injury	Shuttling from the cytosol to nucleus in bronchial epithelial cells	Interacting with p65 in the cytosol and translocating to the nucleus, eventually triggering an inflammatory response	Non-enzymatic activity	[95]
Myocardial infarction	Not detected	Decreasing miR-19a-3p and elevating CDK2, leading to myocardial infarction	Enzymatic activity	[67]
Myocardial infarction	Nucleus in macrophage	Forming HDAC3-NCoR1 repressor complex and inhibiting *Arg1* expression independent of its enzymatical activity	Non-enzymatic activity	[69]
Diabetic myocardial IRI	Nucleus in cardiomyocytes	Regulating circadian gene oscillations to trigger mitophagy dysfunction and myocardial IRI	Non-enzymatic activity	[15]
Diabetic endothelial dysfunction	Cytosol in endothelial cells	Inhibiting Nrf2 signaling through the modulation of Keap1 and Nox4	Enzymatic activity	[74]
Renal fibrosis	The nucleus of renal tubular cells	Inhibiting Klotho transcription and mediating myofibroblast transdifferentiation by decreasing acetylations of H3K4, H3K9, and H4K5 thus promotes renal fibrosis	Enzymatic activity	[89]
Diabetic renal damage	Not detected	Epigenetically modulating miR-10a, subsequently affecting CREB1 and fibronectin formation	Enzymatic activity	[92]
Diabetic hepatic damage	Mainly concentrating in the cytosol in hepatocyte	Decreasing Nrf2 activity by inhibiting miR-200a expression with a concomitant increase in Keap1 to block hepatic FGF21 synthesis.	Enzymatic activity	[107]
Diet-induced obesity	The nucleus in intestinal epithelial cells	Regulating expression of microbiota-dependent metabolic pathways including *Chka, Mttp, Apoa1, and Pck1*, thus triggering diet-induced obesity	Enzymatic activity	[108]
Diabetes mellitus	Both nucleus and the cytosol in β-cells	Affecting insulin secretion, glucose tolerance, lipotoxicity, insulin resistance, and inflammation	Enzymatic activity	[116, 117, 134]

## The emerging roles of HDAC3 in solid organ injury

### Brain

Ischemic stroke is a potentially deadly cardiovascular disease causing significant morbidity and mortality worldwide. Ischemic stroke is usually triggered by the thrombus in the cerebral vasculature. Persistent occlusion in cerebral vasculature may hinder the supply of oxygen and glucose in the local brain tissue, eventually causing neuroinflammation, neuronal cell death, and secondary tissue injury during cerebral hypoperfusion and reperfusion [52, 53]. Type-1 interferons (IFNs) are pleiotropic cytokines regulating the expression of proinflammatory genes and orchestrating innate immune responses [54]. Hence, targeting IFNs or their upstream regulators may help prevent occlusion-induced brain injury [55, 56]. The cyclic GMP-AMP (cGAMP) synthase (cGAS)-stimulator of interferon genes (STING) pathway is a key regulator of the IFN pathway and innate immunity in response to double-stranded DNA (dsDNA) [57]. Microglia are innate immune cells residing in the central nervous system and serve as the principal effector cells contributing to neuroinflammation and brain injury caused by IRI. Conditional knockout of cGAS in microglia significantly relieved cerebral IRI. Mice with microglia-specific deletion of *Hdac3* displayed low expression of cGAS at the mRNA and protein levels, as well as decreased levels of STING and IFN-γ. Mechanistically, HDAC3 deacetylates p65 at K122 and promotes the nuclear accumulation of p65, which regulates the transcription of cGAS [37]. HDAC3 inhibition using RGFP966 dampened the activation of melanoma 2 (AIM2) inflammasome in microglia, preventing ischemic brain injury [58]. Proteomic analysis revealed another mechanism of how HADC3 contributes to microglial injury. Specifically, genes involved in the toll-like receptor (TLR) pathway and STAT3/5 pathway were found to be differentially expressed between HDAC3 inhibitor-treated and untreated lipopolysaccharide (LPS)-exposed primary microglia [59]. Diabetes mellitus significantly increases the risk of cerebral vessel occlusion and is one of the predominant risk factors for ischemic stroke [60]. HDAC3 inhibition mitigated cerebral IRI in diabetic mice by upregulating the brain and muscle Arnt-like 1 (Bmal1) gene [61]. However, whether the ability of HDAC3 to regulate Bmal1 expression depends on histone deacetylation remains unclear.

**Fig. 4:**
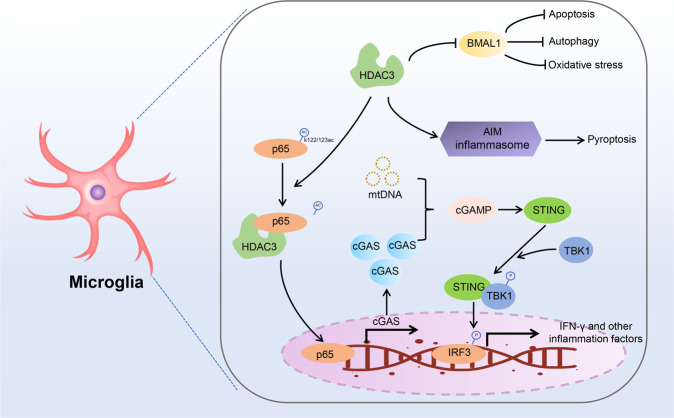
The role of HDAC3 in brain injury. HDAC3 promotes neuronal cell death via multiple mechanisms. HDAC3 is upregulated in microglia after ischemia stimulation, deacetylating p65 at K122. Deacetylated p65 translocates into the nucleus activating the transcription of cGAS. Ischemia-induced mitochondrial DNA is recognized by cGAS, which activates the microglial cGAS-STING-IRF3 pathway and promotes neuroinflammation. In addition, HDAC3 inhibits the activation of the AIM inflammasome and induces pyroptosis. HDAC3 also induces apoptosis, autophagy, and oxidative stress by inhibiting the expression of BMAL1.

Alzheimer’s disease (AD) is a leading cause of age-related neuronal degeneration. AD is characterized by the deposition of amyloid-beta (Aβ) senile plaques in the extracellular milieu, deposition neurofibrillary tangles encompassing acetylated and hyperphosphorylated tau, and synapse dysfunction [62]. Recently, histone deacetylation at specific lysine residues has been implicated in AD, and nonspecific HDAC inhibitors have been used in vitro and in vivo to alleviate AD [63]. However, although these nonselective HDAC inhibitors have displayed promising anti-AD effects, they may cause various side effects. Interestingly, HDAC3 silencing or inhibition increased the acetylation of histones H3 and H4, as well as reduced the phosphorylation at Thr^181^, Ser^202^, and Ser^396^ and acetylation of tau protein. In addition, HDAC3 silencing or inhibition decreased Aβ1–42 accumulation and β-secretase-mediate cleavage of the amyloid precursor protein in HEK/APPsw cells, hinting that HDAC3 may act as a critical regulator of AD-associated brain injury ^36^. Moreover, HDAC3 inhibition inhibited the oxidation of proteins, DNA, RNA, and lipids in the hippocampi of mice with AD by inactivating the c-Abl/MST1/YAP signaling pathway [64]. Collectively, the findings of these studies suggest that HDAC3 plays a crucial role in brain injury by regulating innate immunity, inflammation, and the biological clock (Fig. 4). In addition, we found that HDAC3 promotes the deacetylation of both non-histones and histones in the brain, indirectly or directly affecting the expression of target genes. However, considering that HDAC3 possesses both enzymatic and non-enzymatic activities, how HDAC3 impacts the target proteins has not been comprehensively investigated yet.

### Heart

Myocardial infarction (MI) is a significant global health burden. Ischemic stress due to coronary artery obstruction impedes the supply of oxygen and nutrients to the myocardium, causing tissue injury and cardiomyocyte death [65]. Cyclin-dependent kinase 2 (CDK2) is a serine/threonine kinase, and its dysregulated activation has been associated with MI and heart failure [66]. HDAC3 in cardiomyocytes can induce cardiac dysfunction and heart failure. RGFP966 treatment in mice with MI alleviated oxidative stress and cardiac injury. In addition, RGFP966 decreased the expression of CDK2 in the myocardium by promoting miR-19a-3p expression. However, how HDAC3 regulates the miR-19a-3p expression remains unclear [67]. During MI, cardiomyocyte death activates acute inflammation through the recruitment of macrophages to the infarcted myocardium to prevent further loss of cardiomyocytes, fibrosis, and tissue damage [68]. The Hippo signaling pathway regulates cellular homeostasis, immunity, heart development, and regeneration. The transcription factors yes-associated protein (YAP) and transcriptional co-activator with PDZ-binding motif (TAZ) regulate the innate and adaptive immunity in response to Hippo pathway activation [68]. After MI, YAP/TAZ deficiency modulates the macrophage phenotype, promoting tissue damage repair and maintaining cardiac function. Mechanistically, YAP/TAZ in reparative macrophages inhibits the transcriptional activation of *Arg1* by binding to its promoter and interacting with the HDAC3-NCoR1 repressor complex. This inhibition of *Arg1* expression and the reprogramming of macrophages is independent of the deacetylation activity of HDAC3 [69]. Notably, HDAC3 knockout did not change the levels of H3K9Ac and H3K27Ac in the HDAC3-binding sites in the *Arg1* promoter, suggesting that HDAC3-mediated deacetylation is not essential for the inhibition of *Arg1* expression [70].

Diabetes mellitus increases the risk of MI. Patients with diabetes mellitus and MI tend to have larger infarcts in the myocardium and are at a higher risk of heart failure [71]. Circadian clock genes may regulate MI and diabetes mellitus by modulating the time-of-day dependence of cardiomyocytes [72]. HDAC3 is a vital regulator of the circadian rhythm and may suppress the expression of BMAL1 by activating the expression of the clock gene Rev-erbα [73]. In diabetic rats with or without MI, the expression levels of HDAC3 were constant. However, ischemia-reperfusion significantly increased the expression levels of HDAC3 in diabetic rats. Intriguingly, the levels of Rev-erbα and BMAL1 displayed opposite rhythm after ischemia-reperfusion stimulation in diabetic rats. Subsequent studies showed that HDAC3 aggravated diabetic MI in rats by altering the oscillations of circadian genes, thereby causing mitophagy dysfunction in cardiomyocytes [15]. Interestingly, hyperglycemia significantly increased HDAC3 protein levels. HDAC3 inhibition relieved endothelial injury and dysfunction induced by type 2 diabetes mellitus by blocking the interaction of Keap1 and Nrf2 in a Nox4-dependent manner [74].

Heart failure is the end stage of various cardiovascular diseases and represents a critical healthcare burden. Several mechanism-based therapies for heart failure have been developed recently [75, 76]. The reduced expression level of HDAC3 was associated with better cardiac function in mice with heart failure [77]. In addition, HDAC3 has been shown to promote heart failure and dietary death by exacerbating metabolic disturbances in mitochondria in the cardiomyocytes of mice fed with a high-fat diet [78]. Mechanically, HDAC3 inhibition prevented heart failure by inhibiting miR-18a-targeted adrenergic-receptor β3 [79]. HDAC3 expression and activation in cardiomyocytes were also regulated by Ca^2+^/calmodulin-dependent kinase II (CaMKII). Specifically, CaMKII increased HDAC3 expression levels and enhanced the deacetylase activity of HDAC1 and HDAC3. However, CaMKII hyperactivity-induced heart failure could be reversed by class I HDAC inhibitors [80]. Endothelial dysfunction is another critical mechanism contributing to heart failure, diabetic cardiomyopathy, and diabetic microvascular disease. In high glucose-treated endothelial cells, β-hydroxybutyrate blocked the binding and colocalization of HDAC3 to β-catenin via increasing H3K14ac levels, thereby increasing the expression of claudin-5 and relieving cardiac microvascular hyperpermeability in diabetic rats [81]. However, the mechanisms underlying β-hydroxybutyrate-mediated H3K14ac regulation in endothelial cells need to be further investigated. These studies have provided strong evidence supporting that HDAC3 is activated in the myocardium during acute and chronic cardiac injury, aggravating the cardiac injury by reprogramming macrophages, regulating the expression of clock genes, activating adrenergic receptors, and increasing microvascular endothelial hyperpermeability.

### Kidneys

**Fig. 5:**
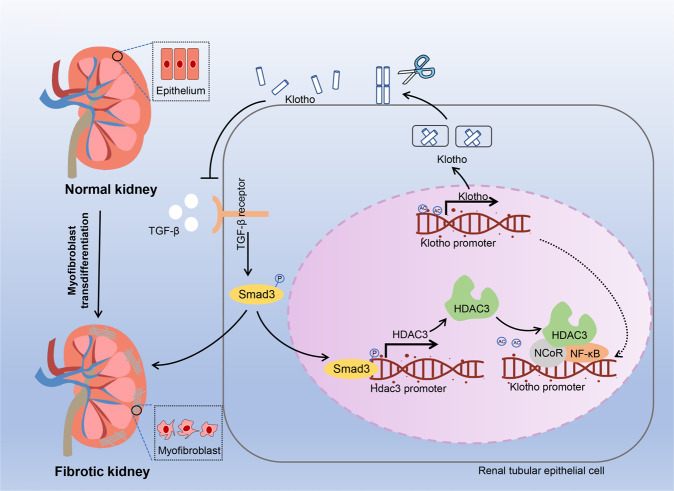
Scheme of HDAC3-mediated renal fibrosis. Renal injury enhances the production of TGF-β, which promotes myofibroblast differentiation and activates *Hdac3* transcription and Smad signaling. Subsequently, HDAC3, together with NF-κB and NCoR, bind to and deacetylate the Klotho promoter, downregulating Klotho and exacerbating renal fibrosis. HDAC3 inhibition preserves the expression of Klotho, inhibiting the TGF-β receptor and alleviating renal fibrosis.

Renal fibrosis is a typical pathohistological characteristic of renal aging and chronic renal injury. Renal injury can be triggered by various etiologies and is characterized by the transdifferentiation of injured renal cells to myofibroblasts [82]. In renal fibrosis, transdifferentiated myofibroblasts secrete extracellular matrix (ECM) proteins, which gradually deteriorate renal structure and function [83]. Although the precise mechanisms underlying renal fibrosis remain primarily unclear, accumulating evidence suggests the critical role of the transforming growth factor-β (TGF-β)/Smad pathway in this process. Specifically, TGF-β1 promotes the recruitment of activated serine kinases, which phosphorylate Smad2 and Smad3. Phosphorylated Smad2 and Smad3 form a complex with Smad4 and translocate into the nucleus, inducing the expression of profibrotic genes [84, 85]. Klotho is an anti-fibrosis and anti-aging protein enriched in the renal tube epithelium. It can be found in a soluble or membrane-bound form [86]. Klotho-deficient mice spontaneously developed renal fibrosis and displayed aging phenotypes in various organs. By directly binding to TGF-β receptor and Wnt, Klotho inhibits the profibrotic TGF-β/Smad and Wnt/β-catenin pathways [87, 88]. In mice with renal fibrosis induced by unilateral ureter obstruction, the expression levels of HDAC3 in the nucleus of renal tubular cells were significantly elevated in a TGF-β/Smad signaling-dependent manner. The renal fibrosis-induced HDAC3 upregulation was reversed by the selective inhibition of Smad3 phosphorylation. HDAC3 accumulation in the nucleus further enhanced the transcriptional repression of Klotho. By interacting with NCoR and NF-κB, HDAC3 triggered myofibroblast transdifferentiation and aggravated renal fibrosis (Fig. 5) [85, 89]. The pan-HDAC inhibitor trichostatin has been reported to block TGF-β1-induced epithelial-mesenchymal transition in human renal epithelial cells and IRI-induced renal fibrosis [90][25]. FK228, a class I HDACs inhibitor predominately targeting HDAC1 and HDAC2, also suppressed the activation and proliferation of renal fibroblasts by enhancing histone H3 acetylation partially through the Smad pathway. Therefore, class I HDACs seem to prevent renal fibrosis by similar mechanisms.

Renal IRI is common during renal transplantation, cardiovascular surgery, trauma, and endovascular procedures. TSA pretreatment early after renal ischemic injury protected renal function and prevented renal fibrosis by upregulating miR-21. Notably, HDAC6 inhibition exhibited no significant effects on renal IRI tolerance, suggesting that class II HDAC elimination is unlikely to contribute to renal IRI tolerance [90]. Diabetic nephropathy is a chronic renal injury in patients with diabetes mellitus. Our poor understanding of its pathogenesis makes the treatment of diabetic nephropathy challenging [91]. In mice fed a high-fat diet and treated with a low dose of streptozotocin, HDAC3 downregulated miR-10a and upregulated cAMP response element-binding protein 1 (CREB1), thereby promoting kidney injury [92]. However, how HDAC3 epigenetically modifies the expression of miR-10a remains unknown. These data together indicate that both acute and chronic kidney injury is closely associated with HDAC3 overexpression, suggesting the inhibition of HDAC3 as a promising therapeutic strategy to protect renal function.

### Lungs

Acute lung injury is characterized by excessive damage to alveolar epithelial cells and capillary endothelial cells due to infection, ischemia-reperfusion, trauma, blood transfusion, and pulmonary embolism. Lung injury eventually causes refractory hypoxemia and acute respiratory distress syndrome (ARDS) [93, 94]. During sepsis-induced acute lung injury, LPS can bind to Toll-like receptor-4 (TLR-4) and its co-receptor cluster of differentiation 14 (CD14), causing neutrophil accumulation in the alveolar space and interstitial tissue, lung parenchymal damage, increased vascular permeability, and aggravation of pulmonary edema. Furthermore, LPS-induced TLR4 activation promoted the translocation of HDAC3 and NF-κB into the nucleus in alveolar epithelial cells; nimbolide could reverse this effect [95]. In addition, HDAC3 inhibition suppressed the expression of various proinflammatory cytokines (e.g., IL-1β, IL-6, and IL-12β) in macrophages by inhibiting NF-κB, thereby preventing acute lung injury [96]. A recent study showed that LPS stimulation did not change the ability of HDAC3 to bind near deacetylation-dependent genes but increased the binding of HDAC3 to transcription start sites in deacetylation-independent genes. In bone marrow-derived macrophages, HDAC3 promoted the expression of proinflammatory cytokines by binding to ATF2-binding sites independently of the NCoR1/2 complex. On the contrary, ATF3-bindings sites were preferentially related with the deacetylase activity of HDAC3, the activation of which inhibited the TLR signaling pathway and inflammation in macrophages [97]. Thus, during macrophage activation or acute lung injury, HDAC3 plays multifaceted roles in the expression of proinflammatory cytokines, which was determined by the enzymatic activity of HDAC3.

PM2.5 is a type of air particle with a diameter of ≤2.5 µm. Exposure to PM2.5 poses a great threat to our health [98]. PM2.5 can invade small airways and interfere with pulmonary gas exchange, eventually causing chronic lung injury and lung cancers [99, 100]. Mechanistically, PM2.5 causes ultrastructural alterations in mitochondria and membrane lysis in alveolar epithelial cells, in addition to inducing DNA damage and the production of reactive oxygen species (ROS) [101–103]. Furthermore, accumulating evidence suggests that PM2.5 can also activate TLR4, TLR4/NF-κB, and TGF-β/Smads, causing pulmonary inflammation and fibrosis [98, 104]. In this regard, HDAC3 deficiency remitted PM2.5-induced damage and inflammation in lung epithelial cells by inhibiting TGF-β/Smad3 and NF-κB signaling [98].

Although HDAC3 inhibition may prevent lung injury by repressing inflammation, particular attention should be paid to the development of HDAC3-targeting agents as the enzymatic activity of HDAC3 can regulate inflammation in opposing ways.

### Liver

Long-term high blood glucose or a high-fat diet can result in liver damage due to increased lipid peroxidation. In turn, chronic liver damage can cause multiple vascular complications, including atherosclerosis [105]. Fibroblast growth factor 21 (FGF21) is a newly identified member of the FGF family regulating glucose and lipid metabolism in the liver [106]. HDAC3 inhibition using RGFP966 mitigated diabetes-induced inflammation, aortic fibrosis, and pathological liver injury in mice with type 1 diabetes mellitus. In addition, HDAC3 inhibition suppressed Keap1 translation by upregulating miR-200a. Keap1 downregulation enhanced the transcription of Nrf2 target genes, including heme oxygenase-1 (HO-1), catalase, and nicotinamide adenine dinucleotide phosphate (NADPH) quinone oxidoreductase (NQO1). These antioxidants increased the level of FGF21 in the serum and alleviated oxidative stress in the liver [107]. In mice with type 2 diabetes mellitus, intestinal epithelial cell-specific disruption of Hdac3 prevented obesity and dysregulation of glucose metabolism. Consistently, HDAC3 inhibition prevented hepatic injury and fat deposition [107]. Clinical findings also indicated that HDAC3 expression levels in pediatric patients were positively associated with bodyweight [108]. HDAC3 interacts with various nuclear proteins in hepatocytes. Particularly, HDAC3 was found to interact with the prospero-related homeobox 1 (PROX1) in a hepatocyte nuclear factor 4α (HNF4α)-dependent manner [109]. PROX1 is a highly conserved transcription factor essential for the development of multiple organs in vertebrates [110]. Although *Prox1* knockdown in adult hepatocytes did not affect the expression of HDAC3, it increased the hepatic levels of triglycerides. These data suggest that the interaction between PROX1 and HDAC3 is essential for maintaining hepatic lipid homeostasis and lipid metabolism [109]. Hence, HDAC3 acts as a double-edged sword in hepatic injury. Both overexpression and downregulation of HDAC3 trigger hepatic injury by disturbing metabolic balance. The mechanisms of how HDAC3 regulates type 1 and type 2 diabetes mellitus-induced hepatic injury are different, although HDAC3 has been implicated in both diseases.

### Pancreas

Type 1 diabetes mellitus is a chronic autoimmune disease caused by the immune-mediated destruction of insulin-producing β-cells in pancreatic islets, leading to insulin deficiency. By contrast, type 2 diabetes mellitus is characterized by progressive loss of β-cell insulin secretion, which usually occurs in the context of insulin resistance. Loss of functional β-cell mass, whether type 1 diabetes mellitus or type 2 diabetes mellitus, serves as the core mechanism in both diseases. Normoglycemia can be preserved as long as β-cells possess the ability to compensate [111, 112]. B lymphocytes and T lymphocytes participate in the destruction of β-cells and the loss of self-tolerance in patients with type 1 diabetes mellitus [113]. HDAC3 inhibited apoptosis in peripheral blood mononuclear cells by downregulating miR-296-5p and upregulating Bcl-xl, thereby aggravating type 1 diabetes mellitus [114]. Conversely, HDAC3 was found to be downregulated in the pancreas of children with type 1 diabetes mellitus. In the rat β-cell line INS-1, HDAC1 or HDAC3 knockdown decreased iNos mRNA levels in response to cytokines. However, only HDAC1 knockdown restored insulin secretion in cytokine-treated β-cells [115]. HDAC3 inhibition also inhibited apoptosis, caspase 3 activations, and Erk1/2 phosphorylation in palmitic acid-treated NIT-1 cells, thereby restoring glucose tolerance [116]. In streptozotocin-induced type 1 diabetic mice, HDAC3 inhibition exerted a hypoglycemic effect and improved the morphology of islets and the function of β-cells [116].

MS-275, a nonspecific inhibitor of HDAC1 and HDAC3, potentiated insulin secretion in the islets of rats with type 2 diabetes mellitus. Bioinformatics analysis revealed that the differentially expressed genes between normal islets and MS-275-treated islets were enriched in calcium, cAMP, MAPK, PI3K-Akt, and Rap1 signaling. However, the genes dysregulated by MS-275 in rat islets were not involved in glucose oxidation [117]. Consistently, SJ Lee et al. found that HDAC3 levels were elevated in palmitic acid-treated C_2_C_12_ myotube. MS-275 pretreatment or HDAC3 knockdown dramatically alleviated lipotoxicity and protected against palmitic acid-induced insulin resistance and inflammation. In addition, MS-275 improved mitochondrial function in palmitic acid-treated C_2_C_12_ myotubes by enhancing mitochondrial fatty acid oxidation and upregulating mitochondrial transcription factor A, peroxisome proliferator activator receptor γ (PPAR-γ)-co-activator 1α (PGC1α), 3-hydroxy acyl CoA dehydrogenase, and enoyl-CoA hydratase [118]. HDAC3 inhibition also exerted an anti-apoptotic effect in β-cells under glucolipotoxic conditions by alleviating endoplasmic reticulum stress [119]. Thus, HDAC3 can affect insulin secretion, glucose tolerance, lipotoxicity, insulin resistance, and inflammation in pancreatic cells via multiple mechanisms. Therefore, pharmacological inhibition of HDAC3 may serve as a novel therapeutic strategy to treat patients with diabetes mellitus. However, more studies are required to uncover the different roles of HDAC3 in type 1 and type 2 diabetes mellitus.

## Selective HDAC3 inhibitors

Over the last 20 years, several HDAC3 inhibitors have been developed and tested for the treatment of various diseases. Thus far, the U.S. Food and Drug Administration (FDA) has approved six HDAC inhibitors, namely belinostat, vorinostat, romidepsin, chidamide, pracinostat, and panobinostat. Most of these inhibitors are used as anticancer agents [120]. As all class I HDACs have a zinc ion in the active site, small molecule inhibitors typically possess the same zinc-binding group. In addition, most HDAC inhibitors possess a linker connecting the zinc-binding group to a capping group to mimic the lysine alkyl side chain. The most well-known zinc-binding group involves *o*-aminoanilides and hydroxamic acids. Entinostat (MS-275) and other selective class I inhibitors are based on *o*-aminoanilides. The selective HDAC3 inhibitor RGFP966 was developed through the modification of *o*-aminoanilides. PD106 and BRD3308 are also selective HDAC3 inhibitors used for the treatment of Friedreich’s ataxia, diabetes mellitus, and HIV infection [121]. Considering the potential of the *o*-aminoanilide scaffold in the development of selective HDAC3 inhibitors, many derivatives were synthesized based on this scaffold. In addition, some natural compounds can also regulate the expression or activity of HDAC3, exerting protective effects in various diseases in animal and cell models. For instance, juglanin prevents high-fat diet-induced renal injury by blocking the nuclear translocation of HDAC3 and NF-κB and thereby inhibiting inflammation and dyslipidemia [122]. miRNAs also play critical roles in nerve injury by targeting HDAC3 [123, 124]. The most common HDAC3 inhibitors and their roles in solid organ injury are summarized in Table 2. Most of the synthesized HDAC3 inhibitors lack specificity, as they partly inhibit other HDACs because of the high structural similarity of HDACs. Nearly all selective HDAC3 inhibitors are *o*-aminoanilide derivatives, the selectivity of which is mainly accessed through testing their IC_50s_ [125]. However, this test method is not accurate. Alternatively, the *K*_*i*_ value of the *o*-aminoanilides can be determined by testing the *k*_on_ and *k*_off_ values of the inhibitors directly.

**Table 2:** Common HDAC3 inhibitors and their role in solid organ injury.

Inhibitor	Models	Key molecular signaling	Major outcome	Reference
RGFP966	Ischemic brain damage	Acetylating and dephosphorylating STAT1, inhibiting the AIM2 inflammasome	Protecting against inflammatory response and alleviating ischemic stroke	[58]
	Cerebral IRI in diabetic state	Inhibiting oxidative stress, apoptosis, and autophagy by upregulating Bmal1	Decreasing the cerebral infarct volume and inhibiting cytotoxicity	[61]
	Chronic neuropathic injury	Regulating synaptic plasticity	Improving memory impairment	[35]
	Ischemic brain damage	Inhibiting the recruitment of HDAC3 to the promoter regions of *Hspa1a*, *Bcl2l1*, and *Prdx2*	Improving neurotoxicity and neuronal injury	[133]
	Acute lung injury	Repressing Hsp90-dependent RhoA activity	Inhibiting endothelial barrier dysfunction and alleviating LPS-induced lung injury	[135]
	Myocardial IRI in diabetic state	Regulating HDAC3/SIRT1 circuit by regulating Bmal1-mediated autophagy	Alleviating myocardial IRI	[136]
	Myocardial IRI in diabetic state	Activating the Rev-erbα/BMAL1 circadian pathway to inhibit mitophagy	Alleviating myocardial IRI	[15]
	Renal fibrosis	Blocking HDAC3 activity and regulating the expression of Klotho	Alleviating renal fibrosis and improving renal function	[89]
MS275	Seizure-induced brain damage	Downregulating p38 by decreasing histone H3 and H4 methylation and increasing histone H3 and H4 acetylation	Alleviating inflammation and tissue damage	[137]
	Acute lung injury	Maintaining the balance between the anti-inflammatory and proinflammatory IL-10 and IL-12b	Relieving macrophage-induced pulmonary inflammation	[138]
BRD3308	Type 1 diabetes	Decreasing the number of apoptotic β-cells	Inhibiting pancreatic islet infiltration and preventing β-cell death	[139]
MI192	Photothrombotic stroke	Decreasing apoptosis and deacetylation of α-tubulin and upregulating GAP-43 in the cerebral cortex	Eliminating tissue infarct and improving motor activity	[140]
Valproic acid	Sepsis-induced cardiac injury	Increasing histone acetylation in the PTEN promoter and inhibiting the AKT/mTOR pathway	Promoting cardiac autophagy and reducing mitochondrial damage, oxidative stress, and inflammation in cardiac tissues	[141]
	Traumatic brain injury	Inhibiting oxidative stress and autophagy by activating Nrf2/ARE signaling	Reducing microglial activation and inflammation	[142]
Nimbolide	Acute lung injury	Blocking NF-κB and HDAC-3 nuclear translocation mediated by TNF-α	Alleviating oxidative stress, inflammation, and pathological injury	[95]
Betaine	Hypothalamic neural injury	Blocking TLR4/NF-κB pathway activation and repressing HDAC3 expression	Inhibiting hypothalamic astrogliosis and inflammation	[143]
Scriptaid	Ischemic brain damage	Increasing the acetylation of H3 and H4	Decreasing the infarct volume and neuronal degeneration, improving their neurobehavioral dysfunction	[144]
Chrysophanol	Acute lung injury	Promoting HMGB1/HDAC3/NF-κB/p65 complex formation	Relieving lung lesions and enhancing superoxide dismutase levels	[145]
miR-193b-39	Brain injury after subarachnoid hemorrhage	Acetylating p65 by decreasing the expression and activity of HDAC3	Mitigating behavioral impairment, brain edema, blood-brain barrier injury, and neurodegeneration	[146]
miR-494	Ischemic brain damage	Inhibiting the expression of neuronal ataxin-3 and HDAC3 and increasing acetyl-H3K9 levels	Decreasing neuronal apoptosis and infarct size	[124]
miR-19a-3p	Myocardial IRI	Reducing the level of CDK2	Improving cardiac function and attenuating pathological change	[123]

To build up a better picture of the regulatory mechanism of HDAC3, the activation mechanism of HDAC3 should also be paid particular attention. To our knowledge, there are mainly 3 aspects to promote the expression or the activity of HDAC3 at present. On the one hand, HDAC3 could be transcriptionally activated by certain transcription factors including Smad3 including Smad3 through directly binding its promoter [89]. On the other hand, HDAC3 activity could be enhanced via deubiquitinating. For example, HDAC3 activity could be enhanced by ubiquitin-specific protease 38 (USP38) via deubiquitinating. Notably, USP38 knockdown and overexpression could not change the protein level of HDAC3 [126]. In addition, PIWIL2 can also interact with HDAC3, giving rise to the stabilization of HDAC3 from ubiquitin-mediated degradation via competitive relation with E3 ubiquitin ligase Siah2. Meanwhile, PIWIL2 facilitated the interaction between CK2α and HDAC3, thus enhancing HDAC3 activity (Doi: 10.1038/s41419-018-0462-8). Last but not least, the microbiota-derived metabolite [inositol-1,4,5-trisphosphate (InsP3)] could promote epithelial repair by promoting HDAC3 activity, but the detailed mechanism remains unclear [127].

## Clinical value of HDAC3

Till now, a great many clinical studies have also established the clinical position of HDAC3 in solid organ injury. For instance, compared with normal-weight women, obese individuals owned reduced levels of HDAC1, HDAC3, as well as HDAC9 in adipose tissues. Meanwhile, the mRNA levels and activity of these HDACs displayed an inverse correlation with inflammatory markers, obesity indices, and insulin levels, indicating that these HDACs have adverse effects on the development of obesity and diabetes mellitus [128]. One Chinese case-control study also demonstrated that the single nucleotide polymorphism of HDAC3 including rs11741808, rs2547547, and rs2547547 polymorphism was related to type 2 diabetes mellitus [129]. And combined HDAC3 and HDAC9 genes with diabetes mellitus worsen atherosclerosis and resulted in stroke [130, 131]. These clinical studies suggested that the genetic change of HDAC3 is closely associated with the development of diabetes mellitus and cerebrovascular disease. However, few clinical studies have reported the roles of HDAC3 in other types of solid organ injury. Before we reach clinical attempts, we might have to obtain more clinical data on HDAC3 to comprehensively harness the knowledge to benefit humans.

## Future perspective and conclusion

HDAC3 is a unique and important member of the HDAC family. The catalytic activity of HDAC3 mainly depends on the integrity of nuclear receptor co-repressor complexes. In this review, we summarized the enzymatic and non-enzymatic functions of HDCA3. The non-enzymatic functions of HDAC3 should be taken into consideration when developing new HDAC3-targeting strategies. We also briefly discussed the role of HDAC3 in the integration of various signals from the environment to regulate cellular fate, development, metabolism, and energy homeostasis. We also described the role of HDAC3 in the injury of solid organs, including the brain, heart, kidneys, liver, lungs, and pancreas. Although HDAC3 is beneficial under physiological conditions, pathological HDAC3 upregulation is detrimental in the pathogenesis of solid organ injury.

No human disease-causing mutations have been identified in the HDAC3 gene thus far, possible because germline mutations in HDAC3 may be may embryonically lethal in humans. However, numerous single-nucleotide polymorphisms (SNPs) in HDAC3 genes have been reported [130, 132]. We speculate that some SNPs may lie within enhancers and that these SNPs may hinder the recruitment of HDAC3 because of impaired binding of nuclear receptors or transcription factors, dysregulating HDAC3 expression. Another glaring question is how to overcome the high structural similarity of zinc-dependent HDAC isoenzymes to develop specific HDAC3 inhibitors rather than pan-HDAC inhibitors. The biological functions of HDAC3 should also be further explored in future studies.

## Facts

Epigenetic modifications could regulate gene activity, as well as the development of an organism without changing gene sequence.

The reversible acetylation on the N-terminal lysine residues of histone proteins by histone HATs and histone deacetylases HDACs synergistically determine chromatin structure and gene expression.

HDAC3 is closely correlated with acute/chronic injury in the brain, heart, kidney, liver, lung, and pancreas.

## Open questions

What is the physiological function of HDAC3 in development and homeostasis?

What are the precise molecular mechanisms of HDAC3 in solid organ injury?

What are the current situations and challenges in developing novel drugs targeting HDAC3 for solid organ injury?

## Data Availability

No applicable resources were generated or analyzed during this study.
